# Gatherings from Grave Yards, &c. &c.

**Published:** 1840-01-01

**Authors:** 


### GATHERINGS FROM GRAVE YARDS, &c. &c. By *G. A. Walker*, Surgeon. Longman, and Co. 1839.

THE object of the present work is, to open the eyes of the public to the dangerous and disgusting results arising from the present practice of interments in the heart of cities, and in the midst of a dense population; and to endeavour to procure "the entire removal of the dead from the immediate proximity of the living." It cannot be denied that the present manner of burial has become a serious evil, and one well worthy of the attention of the Government; but we are afraid that the custom, which originally arose from the credulous belief of the common people in the powers of the church over souls after death, inducing them to seek a grave in the immediate neighbourhood of some place of worship as a protection against the enemy of mankind, is now too deeply rooted in the human mind to be easily eradicated.

Setting aside, however, the deleterious effects to the human frame from the pestiferous vapours continually exhaling from such enormous masses of putrefying remains, which must exercise a most baneful effect, especially on those living in the more immediate vicinity of these pest-holes,—we think that, if the public attention were to be once fully directed to the disgusting and monstrous practices continually resorted to with regard to the ashes of the dead, in order to make room for fresh supplies, they would be inclined to prefer a sepulture removed from the town, and in which they might rest in peace, to the present revolting practice of huddling up as many human bodies as can possibly be crammed into one narrow space in order that the proprietors of these places may reap as great a harvest as possible. Let us take, for example, one case detailed by our author: he is speaking of the burying-ground underneath Enon Chapel:—

> "This space measures in length 59 feet 3 inches or thereabouts, and in width about 28 feet 8 inches, so that its superficial contents do not exceed 1,700 square feet. Now, allowing for an adult body only twelve feet, and for the young, upon an average, six feet, and supposing an equal number of each to be there deposited, the medium space occupied by each would be nine feet: if, then, every inch of ground were occupied, not more than 189 (say 200 in round numbers) would be placed upon the surface; and admitting (an extravagant admission most certainly) that it were possible to place six tiers of coffins upon each other, the whole space could not contain more than 1,200; and yet it is stated with confidence, and by credible authority, that from 10,000 to 12,000 bodies have been deposited in this very space within the last sixteen years!" 237.

It is not long since an extreme sensation was excited by the greatly exaggerated tales with regard to the practice of exhumation for the purposes of dissection, but we must say that none of these tales could at all equal the wholesale exposure and dismemberment of the dead which must here have been had recourse to.

We will now, however, proceed to a consideration of the book itself.

Mr. Walker devotes the first part of his work to a history of the burial-places of the ancients.

1. *Sepulchres among the Jews.*

According to the Jewish law the touch of a dead body contracted a legal impurity, hence they dreaded all communication with it, so much so, that travellers were even forbidden to walk near the places where the dead had been interred. Every city had its public cemeteries beyond the walls.

2. *Funeral Rites of the Greeks.*

Inhumation appears to have been the most ancient custom among the Greeks, and the practice of conveying the dead to a distance from cities, was sustained both by the laws and by the religious doctrines of this refined nation.

Among the Romans the prohibition of inhumation in towns, was fully established in the law of the twelve tables, "Hominem mortuum in urbe ne sepelito neve urito;" and this law was renewed by all the succeeding forms of government.

Among the early Christians, also, the practice was for a long time zealously observed. The first person to whom they granted the privilege of being buried within the walls of a church was Constantine; after this the practice became more and more common till at last interments within churches was accorded to " Pagans as well as Christians, to the impious and the holy." The prohibition, after Constantine, was renewed in the code of Theodosius, and afterwards in that of Justinian, but though these edicts produced some effect for the time, yet the habit had become so universal, that the efforts to restrain it, made by the decrees of more than twenty councils convened at different periods from the 8th to the 18th century, were of no avail. In 1765, the Parliament of Paris took a stand against the abuses of the present system of interment; in the preamble to their decree on the subject, it was asserted, that " daily complaints are made on the infectious effects of the parish cemeteries, especially when the heats of summer have increased the exhalations; then the air is so corrupted, that the most necessary aliments will only keep a few hours in the neighbouring houses: this proceeds either from the soil being so completely saturated that it cannot retain or absorb any longer the putrescent dissolution, or from the too circumscribed extent of the ground for the number of dead annually interred. The same spot is repeatedly used; and, by the carelessness of those who inter the dead, the graves are, perhaps, often re-opened too soon."

It was not, however, till 1790 that a law was passed, by the National Assembly, commanding all towns and villages to discontinue the use of their old burial-places, and to form others at a distance from their habitations. The cemeteries of Paris are four in number, viz. Père-la-Chaise, Montmartre, Vaugirard, et Mont Parnasse.

" The French Government has therefore shewn itself pre-eminently attentive to the health, and, consequently, to the happiness of its members. Commissions were issued—enquires instituted—laws enacted—royal decrees published, and well arranged plans formed and executed. The remains of those who had long lain mouldering in their tombs have been carefully removed from the interior of cities, and respectfully and securely deposited, and mortuaries have been fixed and consecrated for those who follow so far distant from ' the busy hum of men ' as not to molest or endanger the survivors; whilst in almost every other country the putrefactive process emanating from those who have gone to their last homes is allowed to accumulate in the very midst of the habitations of the living, and to form the nuclei of increase, if not the origin, of the most malignant diseases." 90.

Having thus given an extremely slight sketch of the history of burial places, (for an exceedingly interesting description of which we must refer the reader to the book itself,) we will now follow the author in his description of the state of some of the

### BURYING-PLACES OF THE METROPOLIS.

*Burying Ground, Portugal Street.*—" The soil of this ground is saturated, absolutely saturated, with human putrescence. * * * The effluvia from this ground at certain periods are so offensive, that persons living in Clement's Lane are compelled to keep their windows closed; the walls even of the ground which adjoins the yards of those houses, are frequently seen reeking with fluid, which diffuses a most offensive smell." Typhus fever appears to be very prevalent in the neighbourhood, especially in Clement's Lane, which is absolutely surrounded by burying-grounds.

*Enon Chapel.*—The upper part of this building is devoted to the purposes of public worship, underneath it, is the burying-ground, being separated from it only by a boarded floor. From ten to twelve thousand bodies have been placed here, since its establishment, in pits, the uppermost of which were covered only by a few inches of earth.

" Soon after interments were made, a peculiarly long narrow black fly was observed to crawl out of many of the coffins; this insect, a product of the putrefaction of the bodies, was observed on the following season to be succeeded by another, which had the appearance of a common bug with wings."

A Sunday school is held in the chapel.

" Residents about this spot, in warm and damp weather, have been much annoyed with a peculiarly disgusting smell; and occasionally, when the fire was lighted in a house abutting upon this building, an intolerable stench arose, which it was believed did not proceed from a drain. Vast numbers of rats infest the houses; and meat exposed to this atmosphere, after a few hours, becomes putrid." 156.

*St. Clement's Church, Strand.*—" There is a vault under this church called the '*Rector's Vault,*' the descent into which is in the aisle of the church near the communion table, and when opened the products of the decomposition of animal matter are so powerful, that lighted candles, passed through the opening into the vault, are instantly extinguished; the men at different times employed, have not dared to descend into the vault until two or three days had elapsed after it had been opened, during which period the windows of the church also were opened to admit the perflation of air from the street to occupy the place of the gas emitted;—thus a diluted poison is given in exchange from the dead to the living in one of the most frequented thoroughfares of the metropolis. The other vaults underneath the church are also much crowded with dead. From some cause, at present doubtful, these vaults were discovered to be on fire * upwards of fifty years ago; they continued burning for some days, and many bodies were destroyed." 158.

There was foımerly a well by the side of the church, but the water became so nauseous that it could not be drunk, owing to its becoming impregnated with the putrefying matter with which the ground was charged.

*Drury Lane Burying Ground.*—The ground is now raised to a level with the first-floor windows surrounding the place, in it have been deposited some thousands of bodies.

*Whitechapel Church.*—The burial-ground adjoining the church, placed in the midst of a dense population is so thickly crowded as to present one entire

---

* " This is not a very unusual circumstance; the vaults underneath St. James's Church, Jermyn Street, many years since, were on fire."

mass of human bones and putrefaction. "In digging a foundation for a new wall, the workmen penetrated through a mass of human bones eight or ten feet in thickness; these bones were thrown out and for some time lay exposed to public view, scattered over the ground in a loathsome humid state."—These were afterwards deposited in two or three pits which were filled up to within a few inches of the surface.

*Bunhill Fields, City Road.*—In this burial-ground, occupying about seven acres, more than one hundred thousand interments are supposed to have taken place.

Mr. Walker has given a detailed account of many more of our metropolitan cemeteries; but we think that, from what we have furnished, the reader will be able to form a pretty accurate judgment of the remainder. We will, therefore, extract the account of only one more, to show that this system is not confined to the poorer parts of the town, but extends even to the immediate vicinity of the palace.

"*Buckingham Chapel,* situated in Palace Street, about three minutes' walk from Buckingham Palace. There are two vaults and a burying ground belonging to this chapel; one of the vaults is *underneath very large school-rooms for boys and girls,** and the other is *underneath the chapel;* the entrance to these vaults, is through a trap-door, in the passage, dividing the school-rooms from the chapel; steps lead to the bottom of the building; on the right, is the vault underneath the schools. When I visited this place a body had recently been interred, and the effluvium from it was particularly annoying. The vault is supported on wooden pillars, and there is only one grating, which fronts the street, to admit light and air; the floors of the school-rooms, white-washed on the under surface, form the roof or ceiling of the vault—it is no difficult matter to see the children in the lower school-room from this vault, *as there are apertures in the boards sufficiently large to admit the light from above.* This place is spacious but very low;—the vault on the left, under the chapel, is about the same size as that under the schools, though much lower. I was assured that the ground was so full of bodies, that there was difficulty in allotting a grave: the roof of this vault is formed by the under surface of the floor of the chapel; it is white-washed, the light passes through it; the smell emitted from this place is very offensive. In the vault underneath the chapel there are piles of bodies placed in lead; the upper ones are within a few inches of the wooden floor." 184.

We will not attempt to offer any comments on the facts which have here been detailed; they speak for themselves.

The remainder of the work is principally occupied by the consideration of the ill effects produced on the human constitution by these putrid exhalations; out of the immense number of cases brought forward by our author to prove their dangerous tendencies, we will select two only as illustrations of the results produced during the two most dangerous stages of animal decomposition, which he considers to be,—1st, that which takes place almost immediately after death, and the 2nd, during the extreme degree of putrefaction. As an instance of the evil consequences produced by the exhalation of the gases generated during the first stage, we may quote the following case.

---

* "Some hundreds of children here receive their daily education."

"In the month of June, in the year 1825, a woman died of typhus fever, in the upper part of the house, No. 17, White Horse Yard, Drury Lane; the body which *was buried on the fourth day,* was brought down a narrow staircase: Lewis Swalthey, shoe-maker, then living with his family on the second floor of this house, and now residing at No. 5, Princes Street, Drury Lane, during the time the coffin was placed for a few minutes, in a transverse position, in the doorway of his room in order that it might pass the more easily into the street, was sensible of a most disgusting odour, which escaped from the coffin. He complained almost immediately afterwards *of a peculiar coppery taste,* which he described as being situated at the base of the tongue and posterior part of the throat; in a few hours afterwards, he had at irregular intervals slight sensations of chilliness, which before the next sunset had merged into repeated shiverings of considerable intensity; that evening he was confined to his bed,—he passed through a most severe form of typhus fever; at the expiration of the third week he was removed to the fever hospital—he recovered; he had been in excellent health up to the instant when he was exposed *to this malaria.*" 132.

The effects of the gases produced during the extreme stage of putrefaction are illustrated by the following case.

"My pupil, Mr. J. H. Sutton, accompanied by an individual, for many years occasionally employed in the office of burying the dead, entered the vaults of St. ——— church; a coffin, '*cruelly bloated,*' as one of the grave-diggers expressed it, was chosen for the purpose of obtaining a portion of its gaseous contents. The body, placed upon the top of an immense number of others, had, by the date of the inscription on the plate, been buried upwards of eight years; the instant the small instrument employed had entered the coffin, a most horribly offensive gas issued forth in large quantities. Mr. S. who unfortunately respired a portion of this vapour, would have fallen but for the support afforded by a pillar in the vault; he was instantly seized with a suffocating difficulty of breathing (as though he had respired an atmosphere impregnated with sulphur); he had giddiness, extreme trembling, and prostration of strength; in attempting to leave the vault, he fell from debility; upon reaching the external air, he had nausea, subsequently vomiting, accompanied with frequent flatulent eructations, highly fetid, and having the same character as the gas inspired. He reached home with difficulty, and was confined to his bed during seven days. The pulse, which was scarcely to be recognised at the wrist,—although the heart beat so tumultuously, that its palpitations might be observed beneath the covering of the bed-clothes,—ranged between one hundred and ten and one hundred and twenty-five per minute, during the first three days; for many days after this exposure, his gait was very vascillating." 133.

The remainder of the book is devoted to a general *resumé* of the principal facts and observations contained in the preceding pages.

Having now completed our review of this work, it only remains for us to say, that it will be found well worth the perusal of every person at all interested in the preservation of the health, decency, and cleanliness of the metropolis, and may, perhaps, prove not unacceptable to the general reader, as presenting a complete and curious history of the different modes of interment which have been resorted to among different nations, as well as for its novel description of the burial-places of London, which, we believe, have never, until now, formed the subject of any work.

In taking our final leave, we must, in justice to Mr. Walker, state, that the book is clearly and vividly written, and the author deserves great credit for the industry and zeal which he has displayed in his by no means agreeable researches among the grave-yards; we hope, however, that he may reap a full reward for his labours, by seeing the disgusting nuisance against which he has daclared war, at least mitigated, even if not altogether put a stop to.

